# A case of femoral arteriovenous fistula caused by central venous catheterization under inadequate ultrasound guidance

**DOI:** 10.1186/s40981-018-0167-0

**Published:** 2018-04-04

**Authors:** Daiki Nakashima, Shinobu Yamaguchi, Kumiko Tanabe, Woo Kim, Hiroki Iida

**Affiliations:** Department of Anesthesiology and Pain Medicine, Gifu University Graduate School of Medicine, Gifu, 501-1194 Japan

## To the Editor

### Background

Arteriovenous fistula is a rare but severe complication of central venous catheterization [1, 2]. The use of ultrasound guidance for central venous catheterization is recommended to improve the success rate and reduce complications [3]. However, a safe procedure cannot be performed without adequate knowledge and skill.

## Case presentation

**Fig. 1:**
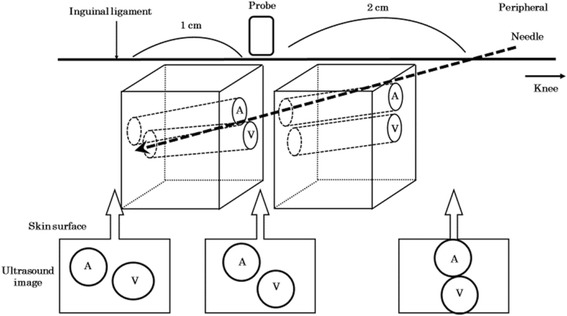
Scheme illustration of the relationship between the needle and ultrasound image. The femoral artery and femoral vein did not overlap at the inguinal ligament level. However, both vessels overlapped toward the periphery. The figures below are ultrasound images at each point. A femoral artery, V femoral vein

A 77-year-old woman was diagnosed with meningioma on the left frontal cerebral falx and scheduled for tumorectomy in the supine position. Under general anesthesia, her right lower extremity was abducted and externally rotated in the supine position for central venous catheterization to the right femoral vein to prepare for intraoperative bleeding. Prescanning for catheterization with ultrasound (SonoSite M – Turbo®, FUJIFILM©) using linear probe was performed. Although the femoral artery and femoral vein did not overlap at the inguinal ligament level, both vessels overlapped toward the periphery. An ultrasound probe was placed 1 cm peripheral from the inguinal ligament, and the skin insertion point of the needle was 3 cm peripheral from the inguinal ligament (Fig. 1). The puncture was performed once using real-time ultrasound guidance (short axis view/out-of-plane approach), and where the needle tip punctured, the target vein was visualized. A central venous double lumen catheter (SMAC Plus®, COVIDIEN™) was inserted smoothly using the Seldinger technique, and reversal of the venous blood was observed.

**Fig. 2:**
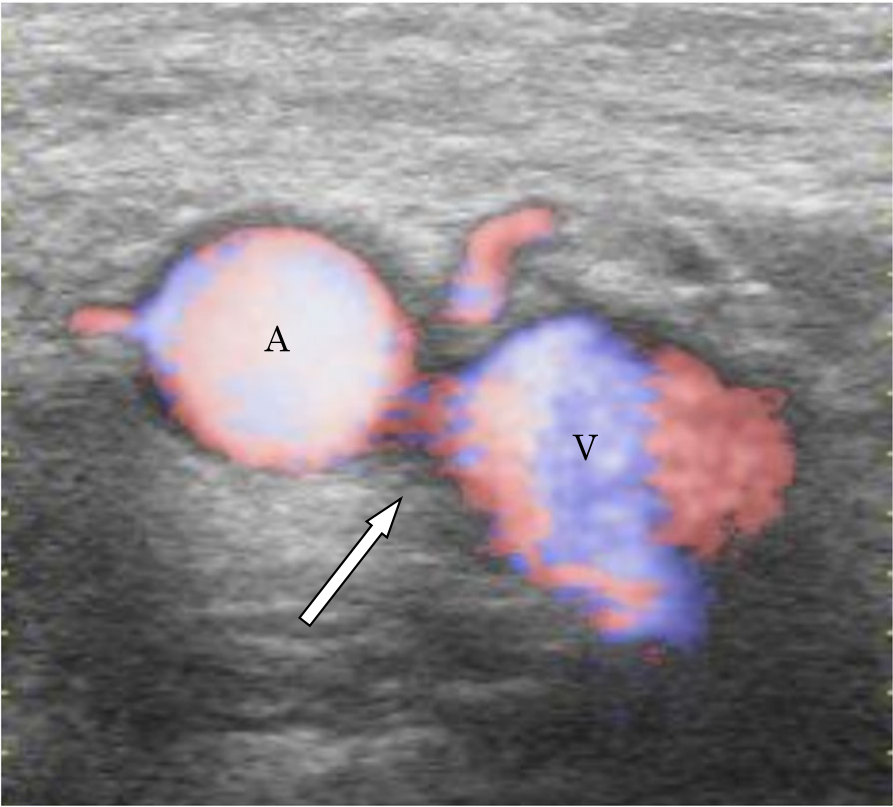
Color Doppler ultrasound findings of an arteriovenous fistula between the right femoral artery and the right femoral vein. A femoral artery, V femoral vein, arrow fistula

When the central venous catheter was removed 6 days after the operation, arterial bleeding was observed. The bleeding stopped by compression with hands. Color Doppler ultrasound and contrast computed tomography detected arteriovenous fistula between the right femoral artery and femoral vein 8 days after the operation (Fig. 2). Angioplasty was performed 15 days after the operation.

## Discussion

In our case, catheterization into the right femoral vein after penetrating the right femoral artery caused femoral arteriovenous fistula. The issue with the manipulation in this case was considered to be the gap between the probe and the needle insertion point of the skin preventing visualization of the needle tip at all times. Therefore, the penetration of the femoral artery by the needle was not recognized. When a central venous catheter is inserted with real-time ultrasound guidance (short axis view/out-of-plane approach), the skin insertion point should be located right next to the probe, and the needle tip should be visualized at all times until the target vein is punctured. In 2014, several key attributes for teaching safe ultrasound-guided central venous catheter insertion were proposed as follows: (1) a curriculum clearly describing the technical approach and cognitive elements required, preferably with video-based procedural examples; (2) hands-on simulation training to develop hand-eye skills; (3) an emphasis on techniques that allow visualization of the needle tip at all times; and (4) insertions supervised by experienced clinicians giving feedback for improvement [4].

## Conclusion

Central venous catheter insertion is an invasive procedure that can lead to severe complications. When a central venous catheter is inserted with or without real-time ultrasound guidance, we must ensure it is adequately inserted.
